# *Bletilla striata* polysaccharides ameliorate metabolic-associated fatty liver disease by decreasing the NLRP3 inflammasome and pyroptosis

**DOI:** 10.3389/fphar.2025.1563275

**Published:** 2025-06-20

**Authors:** Tingting Yu, Juan Xue, Wenqian Tang, Xiaojie Wu, Jun Li, Fan Yang, Lei Luo

**Affiliations:** ^1^ School of Clinical Medical, Hubei University of Chinese Medicine, Wuhan, China; ^2^ Department of Gastroenterology, Hubei Provincial Hospital of Integrated Chinese and Western Medicine, Wuhan, China; ^3^ Department of Health Management Center, Hubei Provincial Hospital of Traditional Chinese Medicine, Wuhan, China

**Keywords:** *Bletilla striata* polysaccharide, metabolic-associated fatty liver disease, nod-like receptor protein 3, pyroptosis, inflammasome

## Abstract

**Background:**

The role of nucleotide-binding oligomerization domain-like receptors containing pyrin domain 3 (NLRP3) inflammasome and pyroptosis in the inflammatory microenvironment of metabolic-associated fatty liver disease (MASLD) has been posited as crucial. *Bletilla striata* polysaccharides (BSPs), extracted from the tubers of *Bletilla striata (Thunb.) Rchb.f.*, exhibit significant anti-inflammatory properties. However, their potential protective effects on MASLD and their role in regulating pyroptosis remain unclear.

**Objectives:**

This study investigates the efficacy of BSP-1, a purified metabolite isolated from crude BSPs, on MASLD by evaluating its ability to modulate the NLRP3/caspase-1/GSDMD signaling pathway.

**Methods:**

To simulate MASLD *in vivo* and *in vitro*, high-fat diet (HFD)-induced rat models and free fatty acid (FFA)-stimulated HepG2 cells were used. Serum indicators and histopathological staining were employed to assess liver injury and lipid deposition. Additionally, enzyme-linked immunosorbent assay (ELISA), immunohistochemistry (IHC), immunofluorescence, real-time quantitative polymerase chain reaction (RT-qPCR), and western blotting (WB) analysis were conducted to examine the NLRP3/caspase-1/GSDMD pathway and related cytokine levels.

**Results:**

BSP-1 significantly ameliorates alanine aminotransferase (ALT), aspartate aminotransferase (AST), total cholesterol (TC), and triglyceride (TG) levels in both rat serum and HepG2 cells. Furthermore, BSP-1 reduces inflammatory factors interleukin (IL)-1β and IL-18, while improving pathological changes in rat liver tissue. Mechanistically, BSP-1 regulates the expression of pyroptosis-related proteins and mRNAs in the NLRP3/caspase-1/GSDMD pathway, thereby protecting against MASLD.

**Discussion:**

BSP-1 may represent a promising therapeutic agent for MASLD treatment by inhibiting the NLRP3/caspase-1/GSDMD signaling pathway.

## 1 Introduction

Metabolic-associated fatty liver disease (MASLD) is a chronic liver condition closely linked to metabolic disturbances. Its histological progression typically advances from simple steatosis to nonalcoholic steatohepatitis, and potentially to liver fibrosis and hepatocellular carcinoma (Kalligeros et al., 2024). Currently, MASLD is recognized as the most prevalent chronic liver disease worldwide, affecting approximately 37.8% of adults globally (Riazi et al., 2022). Due to the stigma associated with the term nonalcoholic fatty liver disease (NAFLD), a series of Delphi surveys resulted in the establishment of new criteria for MASLD that encompass 99% of patients previously diagnosed with NAFLD. Consequently, research data related to NAFLD remain relevant to MASLD (Hagström et al., 2024; Song et al., 2024). As our understanding of MASLD pathogenesis evolves, the widely accepted “multiple hit model” clarifies that after the initial “first hit” of fat accumulation driven by obesity and insulin resistance, the liver undergoes changes influenced by chronic inflammatory pathways, interactions among various organs and tissues (including adipose tissue, pancreas, intestine, cardiovascular system, and kidneys), genetic factors, lifestyle interactions, and broader metabolic dysfunction (Tilg et al., 2021; Targher et al., 2024).

The dynamic balance between cell proliferation and death is a fundamental aspect of both physiological and pathological processes within the body. Pyroptosis, one of the programmed cell death pathways, is characterized by cell swelling, membrane perforation, and the release of cellular contents. In normal physiological contexts, pyroptosis plays a crucial role in the host’s defense against pathogens (Liu et al., 2021). Conversely, in pathological conditions, the excessive cytokine storm and inflammatory response induced by pyroptosis-triggered by inflammasomes and executed by gasdermin proteins-can lead to significant tissue damage and multi-organ dysfunction (Rao et al., 2022). Studies have identified the nucleotide-binding oligomerization domain-like receptors containing pyrin domain 3 (NLRP3) inflammasome, composed of NLRP3, leucine-rich repeats, and a pyrin domain, as a key inflammatory mediator that can be initiated and activated by pathogen-associated molecular patterns (PAMPs), damage-associated molecular patterns (DAMPs), or cytokines involved in immune and inflammatory responses. Activated NLRP3 inflammasomes recruit the downstream molecule caspase-1 through the “bridge” ASC (apoptosis-associated speck-like protein containing a caspase recruitment domain), thereby triggering the maturation of pro-inflammatory cytokines (interleukin (IL)-1β and interleukin (IL)-18) and the processing of gasdermin D (GSDMD) to induce the release of IL-1β and IL-18 and cell pyroptosis (Fu and Wu, 2023). In recent years, with increasing attention to the pathogenesis of MASLD, the pyroptosis-related NLRP3/caspase-1/GSDMD pathway has been recognized as closely associated with the pathogenesis of MASLD (Carvalho Ribeiro and Szabo, 2022). Some natural products, such as penthorum chinense pursh extract, green tea epigallocatechin gallate, have shown to treat MASLD by regulating the pyroptosis-related NLRP3/caspase-1/GSDMD pathway (Luo et al., 2024; Zhang et al., 2025). Therefore, inhibiting the conduction of the pyroptosis pathway may represent a potential therapeutic target for delaying the progression of this disease.

*Bletilla striata (Thunb.) Rchb.f.* tubers (Orchidaceae), a well-known medicinal orchid, has been extensively utilized in traditional Chinese botanical drug for millennia and is reckoned for its efficacy in astringing to stop bleeding, alleviating swelling, and promoting tissue regeneration (He et al., 2017). It exhibits significant health-improving effects in treating digestive tract mucosal injuries, ulcers, hemorrhages, bruises, and burns (Xu et al., 2019). Polysaccharides, the primary bioactive metabolites in *Bletilla striata*, possess various pharmacological properties, including antioxidant, antibacterial, antifibrotic, antiglycation, anticancer, and immunomodulatory activities (Zhu et al., 2023). Meanwhile, BSP has a good regulatory effect on inflammatory response and liver protective effect. BSP can improve liver fibrosis by regulating the TLR2/TLR4-MyD88-NF-κB signaling pathway (Jiang et al., 2023). Additionally, it can also be used as an anti-inflammatory drug to improve acute respiratory distress syndrome (ARDS) by regulating the NLRP3 inflammasome and pyroptosis (Wu et al., 2024). Our previous research has confirmed that crude BSPs enhance the levels of intestinal cellular tight junction proteins, zonula occludens-1 (ZO-1) and occludin both *in vivo* and *in vitro*, while also modulate the expression of inflammatory cytokines (interleukin (IL)-6 and tumor necrosis factor-alpha (TNF-α)) in thioacetamide (TAA)-induced liver cirrhosis rats and lipopolysaccharide (LPS)-induced injury in intestinal epithelial cells, thereby providing a therapeutic strategy for treating inflammation-related diseases induced by gut-derived PAMPs (Luo et al., 2018; Luo, et al., 2019). Hpowever, the potential of BSPs to ameliorate MASLD by regulating the NLRP3 inflammasome and pyroptosis are unknown.

Therefore, in the present investigation, we isolated and purified BSP-1 from crude BSPs using DEAE-Sepharose Fast Flow ion-exchange chromatography and Sephacryl S-100 gel filtration chromatography. We then assessed the impact of BSP-1 on MASLD by evaluating its ability to regulate the expression of pyroptosis-related NLRP3/caspase-1/GSDMD pathway in both high-fat diet (HFD)-induced rat models *in vivo* and free fatty acid (FFA)-induced HepG2 cells *in vitro*. The findings will provide further evidence for the regulative effects and mechanisms of BSP-1 on MASLD.

## 2 Materials and methods

### 2.1 Reagents and antibodies

The HFD was purchased from Wuhan Chunyuhong Experimental Animal Feed Co., Ltd. (56.3% basic feed +20% sucrose +12% lard +5% egg yolk powder +3% milk powder +2% soybean oil +1.5% cholesterol +0.2% sodium cholate). The normal diet was purchased from Henan Huanyu Hekang Biotechnology Co., LTD. (50% corn +22% soybean meal +9% bran +9% flour +7% fish meal +2.5% bone meal +0.5% salt +0.5% vitamin/mineral premix). Kits for measuring alanine aminotransferase (ALT, #C001-a), aspartate aminotransferase (AST, #C002-a), total cholesterol (TC, #C048-a), and triglyceride (TG, #C019-a) levels were provided by Changchun Huili Biotechnology Co., Ltd. (Changchun, China). Enzyme-linked immunosorbent assay (ELISA) kits for IL-18 (#MM-0194R2) and IL-1β (#MM-0047R2) were obtained from Jiangsu Meimian Industrial Company, Ltd. (Jiangsu, China). Anti-β-actin (#66009-1-Ig) was purchased from Wuhan Sanying Biotechnology Co., Ltd. (Wuhan, China). An antibody against NLRP3 (#TU269693) was obtained from Abmart Shanghai Co., Ltd. (Shanghai, China). An antibody against caspase-1 (#AF4005) was obtained from Affinity Biosciences (Cincinnati, OH). An antibody against GSDMD (#10137S) was obtained from ImmunoWay Biotechnology Company (Plano, TX, United States). An antibody against apoptosis-associated speck-like protein containing a caspase recruitment domain (ASC) (#A1170) was obtained from Abclonal (Wuhan, China). HRP-conjugated goat anti-mouse secondary antibody (#BA1051) and HRP-conjugated goat anti-rabbit secondary antibody (#BA1054) were purchased from Wuhan Boster Biotechnology Co., Ltd. (Wuhan, China). Total RNA extraction reagent (#R401-01), HiScript II Q RT SuperMix (#R223-01) and the ChamQ SYBR qPCR Master Mix Kit (#Q311-02) were purchased from Vazyme Biotech Co., Ltd. (Nanjing, China).

### 2.2 Extraction and purification of BSP-1

**FIGURE 1:**
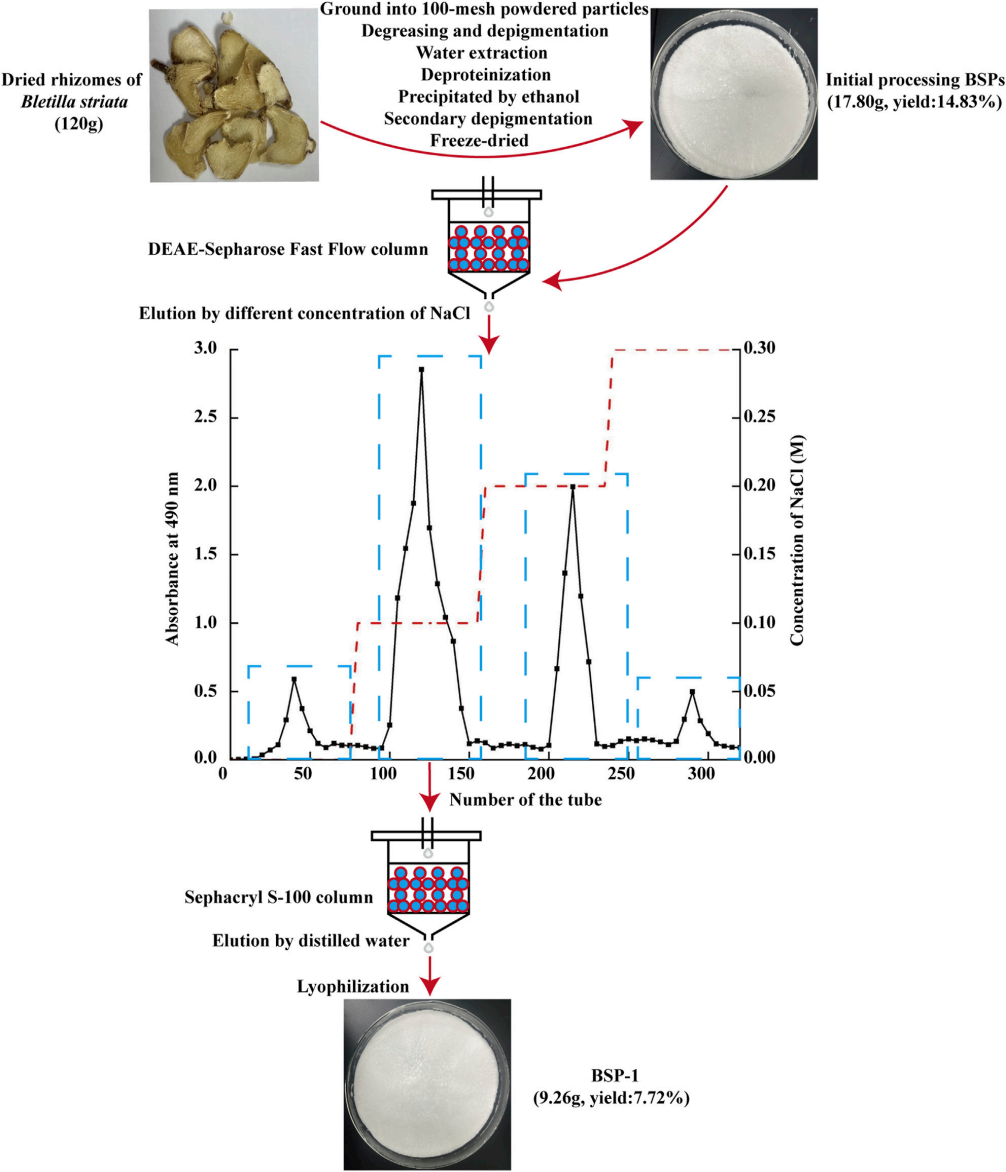
The extraction and purification of BSP-1.

The extraction, isolation, and purification of BSP-1 were performed in accordance with established protocols (Ji et al., 2020). Initially, air-dried *B*. *striata* were obtained from Hubei Provincal Hospital of Traditional Chinese Medicine (Wuhan, China). The material was ground into 100-mesh powdered particles and extracted three times with distilled water at a ratio of 1:15 (W/V) at 90°C for 3 h each time. The three filtrates were collected and mixed, followed by defatting, decolorization, overnight alcohol precipitation at 4°C, and protein removal using the Sevage method. Following these steps, the filtrate was collected through centrifugation, dialyzed with ultrapure water for 48 h, and then lyophilized to yield BSPs. Ultimately, BSP-1 was isolated and purified through DEAE-Sepharose Fast Flow ion exchange chromatography and Sephacryl S-100 gel filtration chromatography, and stored at 4°C (Figure 1).

### 2.3 Characterization of BSP-1

#### 2.3.1 Determination of polysaccharide content and ultraviolet (UV) spectroscopy

The phenol-sulfuric acid method was employed to detect the polysaccharide content in BSP-1, with three replicate measurements taken and their average value calculated. Additionally, BSP-1 at a concentration of 0.1 mg/mL was scanned and assayed for impurity content within the range of 200–400 nm using a UV-Visible spectrophotometer, with distilled water serving as the reference.

#### 2.3.2 Determination of relative molecular weight

To determine the relative molecular weight of BSP-1, 2 mg of BSP-1 was dissolved in 0.2 M NaCl aqueous solution. A 20 μL aliquot of a 5 mg/mL BSP-1 solution was loaded onto a TSK-gel G-3000PWXL stainless steel chromatography column for high-performance gel permeation chromatography analysis. The mobile phase consisted of 0.2 M NaCl aqueous solution at a flow rate of 0.6 mL/min, and the column temperature was maintained at 40°C.

#### 2.3.3 Monosaccharide composition analysis

1 mg of BSP-1 was dissolved in 1 mL of hydrochloric acid-methanol solution, and the mixture was subjected to a constant-temperature metal bath at 80°C under nitrogen gas for 16 h. After the hydrochloric acid-methanol was dried using a nitrogen blow dryer, 1 mL of 2 M trifluoroacetic acid was added, and the reaction was carried out at 120°C for 1 h, followed by drying again. Subsequent to acid hydrolysis, 500 μL of 0.3 M NaOH, 500 μL of 0.5 M 1-phenyl-3-methyl-5-pyrazolone -methanol, 100 μL of 0.3 M HCl, and 700 μL of dichloromethane were added sequentially for dissolution, water bath treatment, extraction, and phase separation. After derivatization of the monosaccharides, high-performance liquid chromatography (HPLC) was employed to detect the monosaccharide composition of BSP-1.

### 2.4 Grouping of experimental animals

Five-week-old male Sprague-Dawley (SD) rats (License No.: SYXK [Hubei] 2023-0067), obtained from the Hubei Provincial Center for Disease Control and Prevention (Wuhan, China), underwent an initial 1-week acclimatization period. The rats were housed in a temperature-controlled room at 25°C ± 1°C with controlled humidity, subjected to a natural light-dark cycle, and had free access to food and water. All experimental protocols used in this study were approved by the Research Ethics Committee of Hubei University of Chinese Medicine (Approval No.: HUCMS00308242) and conducted in accordance with the Guide for the Care and Use of Laboratory Animals.

**FIGURE 2:**
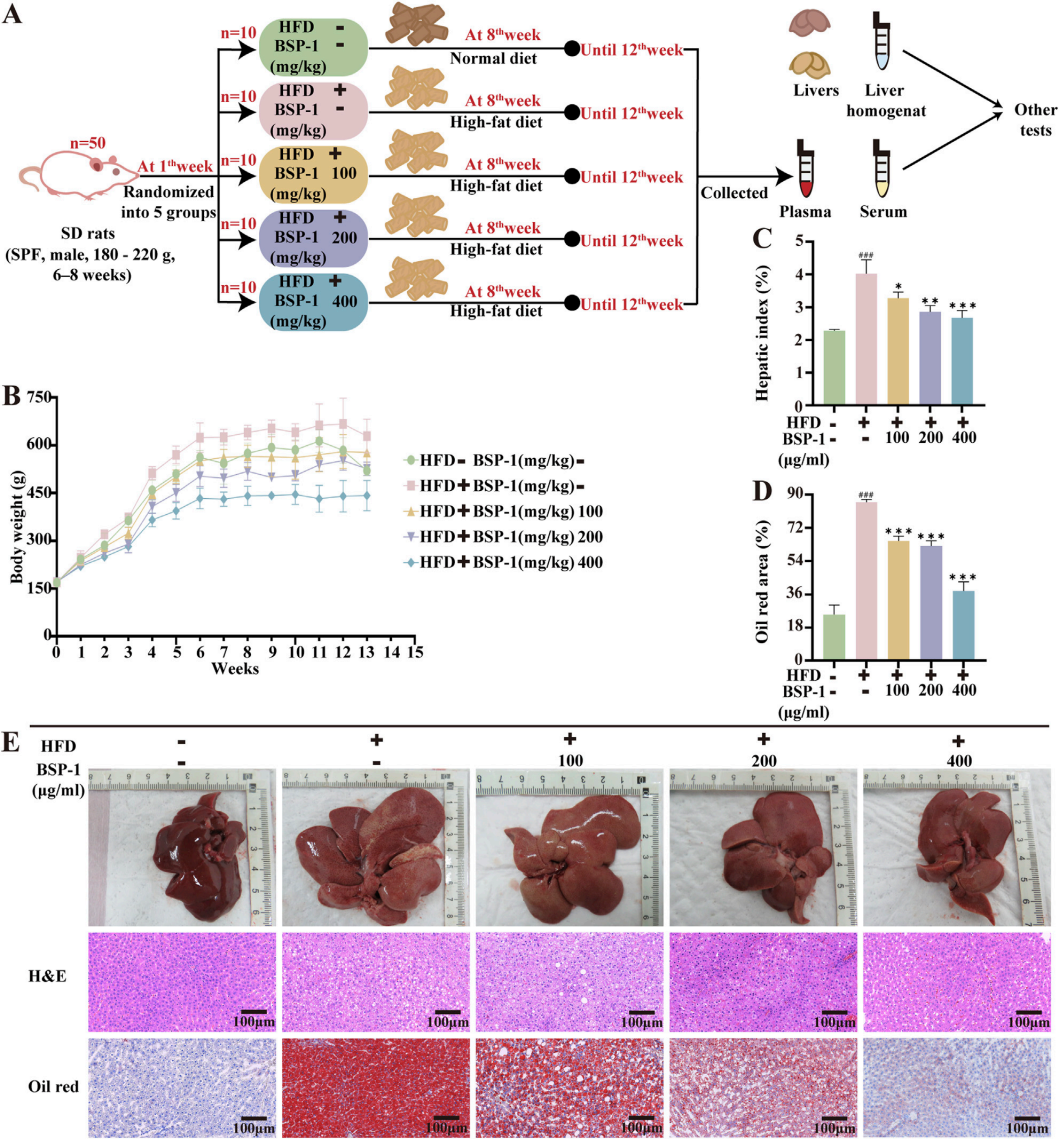
Brief steps of *in vivo* experiments, observation and recording of rat body weight, and pathological changes in liver tissue. **(A)** Grouping of experimental Animals. **(B)** The body weight of rats. **(C)** The hepatic index of rats. **(D)** The oil red area of liver tissue in rats. **(E)** The effect of BSP-1 on liver tissue in rats (anatomical observations and H&E stainning, Oil red stainning, 100 ×). The data are shown as the means ± SDs. ^#^ *p* < 0.05, ^##^ *p* < 0.01, ^###^ *p* < 0.001, vs. the control group; ^*^ *p* < 0.05, ^**^ *p* < 0.01, ^***^ *p* < 0.001, vs. the model group.

The rats were randomly divided into five groups (*n* = 10 rats per group): a normal group, a model group, and low-dose, medium-dose, and high-dose BSP-1 groups. Except for the control group (fed a normal diet), the model group and BSP-1 groups were subjected to a 12-week HFD to induce a model of MASLD (Farage et al., 2023). During the final 4 weeks of the study, the rats in the BSP-1 groups were gavaged with 100 mg/kg/d, 200 mg/kg/d, and 400 mg/kg/d of BSP-1, respectively, while the normal and model groups received an equal volume of saline daily (Qiu et al., 2023). The body weights of the rats were measured weekly during the experimental period. At the end of the experiment, the rats were anesthetized with isoflurane, and blood was collected from the abdominal aorta for serum separation by centrifugation. The liver tissues of the euthanized rats were collected, rinsed with saline, blotted dry, weighed, photographed, and subjected to subsequent analysis. The study procedure is illustrated in Figure 2A.

### 2.5 Histopathological examination of rat liver tissue

Liver tissues from rats in each group were fixed in 4% paraformaldehyde for 24 h, followed by dehydration, paraffin embedding, and sectioning. The slides were then stained with hematoxylin and eosin (H&E) and Oil Red, and observed and photographed under a biological microscope.

### 2.6 Cells and culture

**FIGURE 3:**
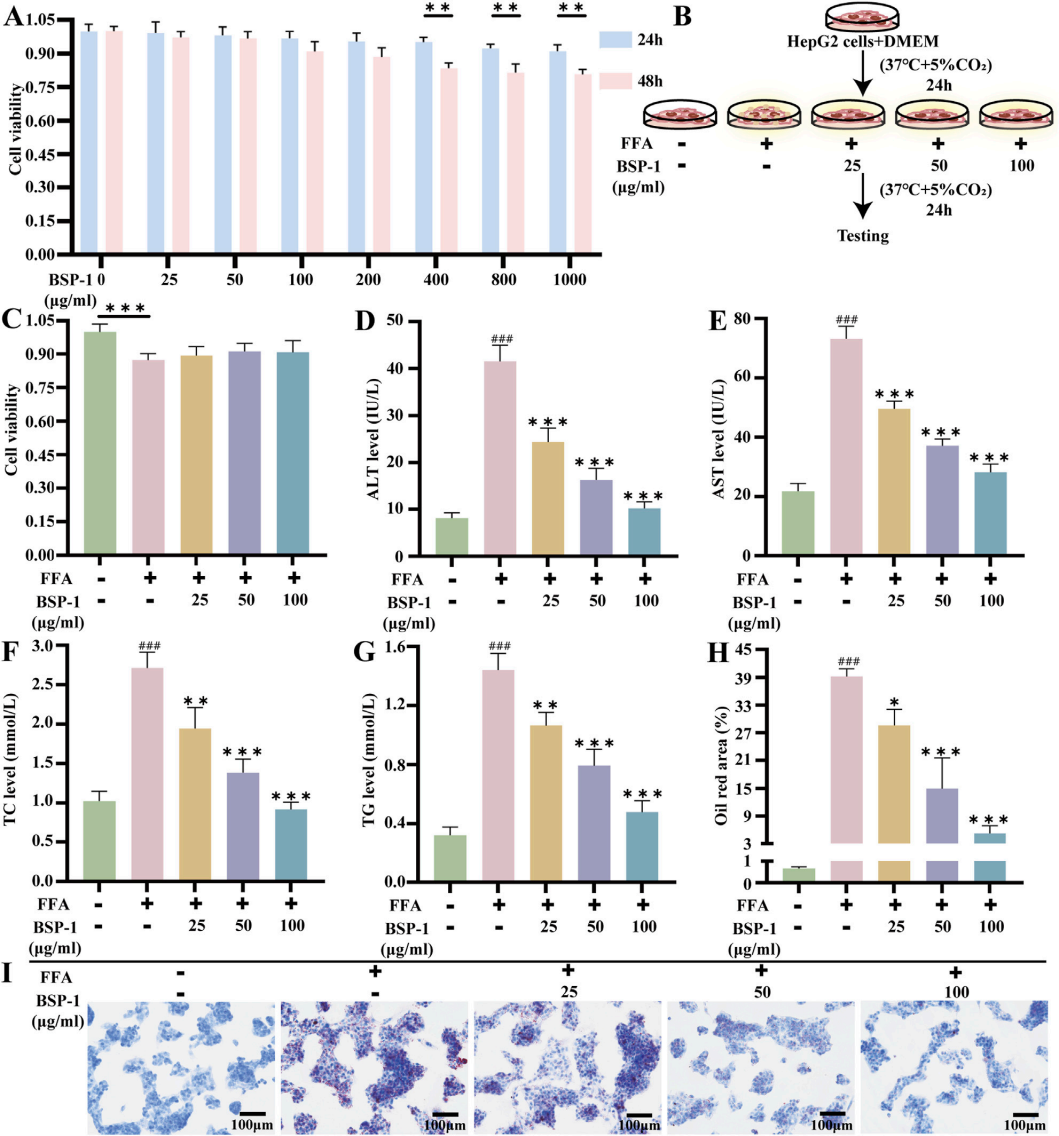
Brief steps *in vitro* experiments, cytotoxicity test of BSP-1, detection of liver function and TC, TG levels, and Oil red staining in HepG2 cells. **(A)** CCK8 assay in HepG2 cells. **(B)** HepG2 cells and culture. **(C)** CCK8 assay in HepG2 cells. The measurement of **(D)** ALT **(E)** AST **(F)** TC **(G)** TG in HepG2 cells. **(H)** The oil red area of liver tissue in HepG2 cells. **(I)** The effect of BSP-1 in HepG2 cells (Oil red stainning, 100 ×). The data are shown as the means ± SDs. ^#^ *p* < 0.05, ^##^ *p* < 0.01, ^###^ *p* < 0.001, vs. the control group; ^*^ *p* < 0.05, ^**^ *p* < 0.01, ^***^ *p* < 0.001, vs. the model group.

HepG2 cells, obtained from Procell of Wuhan (CL-0103) and verified by STR analysis, were cultured in DMEM containing 10% fetal bovine serum and 1% penicillin-streptomycin. Prior to further research, the cells were maintained in a 37°C incubator with 5% CO_2_. The research steps are illustrated in Figure 3B.

### 2.7 CCK8 assay in cells

HepG2 cells were seeded into 96-well plates at a density of 5 × 10^3^ cells per well and cultured in a 37°C incubator with 5% CO_2_ according to different grouping and cell treatments. The groups for assessing the toxicity of BSP-1 on HepG2 cells after 24 h and 48 h of treatment were as follows: normal group, BSP-1 groups (25 μg/mL, 50 μg/mL, 100 μg/mL, 200 μg/mL, 400 μg/mL, 800 μg/mL, 1,000 μg/mL). The groups for assessing the toxicity of BSP-1 on FFA-induced HepG2 cells after 24 h of treatment were as follows: normal group, FFA group, FFA + BSP-1 groups (25 μg/mL, 50 μg/mL, 100 μg/mL). After cell culture, 10 μL of CCK8 was added to each well, and the plates were incubated at 37°C for 1–4 h before measuring the absorbance of each well using a microplate reader.

### 2.8 Measurement of IL-1β and IL-18 in rat serum and HepG2 cells

Rat serum and HepG2 cells from each group, centrifuged at 3,000 rpm for 10 min at 4°C, were used to detect the levels of IL-1β and IL-18 by enzyme-linked immunosorbent assay (ELISA) according to the manufacturer’s instructions.

### 2.9 Measurement of TC and TG contents and liver function in rat serum and HepG2 cells

Rat serum and HepG2 cells from each group, centrifuged at 3,000 rpm for 10 min at 4°C, were used to detect the levels of ALT, AST, TC, and TG in the serum using commercially available kits according to the manufacturer’s instructions.

### 2.10 RT-qPCR analysis of rat liver tissue and HepG2 cells

Total RNA was extracted from rat liver tissue and HepG2 cells using TRIzol reagent according to the manufacturer’s instructions and stored at −80°C until use. The isolated RNA was then quantitated and reverse-transcribed into cDNA using a cDNA reverse transcription kit following the provided protocol. Real-time quantitative polymerase chain reaction (RT-qPCR) was performed using a real-time PCR instrument from Applied Biosystems (California, United States), and the mRNA levels of the target genes were normalized to the relative levels of β-actin using the 2^−ΔΔCT^ method. The primer sequences used in this study are shown in Table 1.

**TABLE 1:** Primer sequence for qRT‒PCR.

Gene	Forward (5′–3′)	Reverse (5′–3′)	Species
NLRP3	CTG​CTG​AAG​TGG​ATC​GAA​GTG	TGC​AAA​AGG​AAG​AAA​CCA​CGT	Rat
GSDMD	CCA​AAG​CCG​GAA​GAA​GAT​GG	ACT​AAA​GTC​ATG​CCG​CCT​CT	Rat
caspase-1	AAC​TGA​ACA​AAG​AAG​GTG​GCG	GCA​GAT​AAT​GAG​GGC​AAG​ACG	Rat
ASC	CTG​TGC​TTA​GAG​ACA​TGG​GCA	GTT​GGT​GGT​CTC​TGC​ACG​AA	Rat
β-actin	TGA​CGT​TGA​CAT​CCG​TAA​AGA​CC	GTG​CTA​GGA​GCC​AGG​GCA​GTA​A	Rat
NLRP3	GTT​TGA​CCC​CGA​TGA​TGA​GC	CTT​GTG​GAT​GGG​TGG​GTT​TG	Homo
GSDMD	AAGACGGTCACCATCCCC	AAG​GTC​CTC​TGC​TTC​TTA​TCC	Homo
caspase-1	GCA​CAC​GTC​TTG​CTC​TCA​TTA	TTC​ACA​TCT​ACG​CTG​TAC​CCC	Homo
ASC	GATCCAGGCCCCTCCTCA	ACC​AGG​TAG​GAC​TGG​GAC​TC	Homo
β-actin	CCC​TGG​AGA​AGA​GCT​ACG​AG	CGT​ACA​GGT​CTT​TGC​GGA​TG	Homo

### 2.11 Western blot (WB) analysis of rat liver tissue and HepG2 cells

Total protein was extracted from rat liver tissue and HepG2 cells using pre-cooled RIPA lysis buffer and quantitated using a BCA protein assay kit. The extracted protein supernatant was boiled for 10 min for denaturation, cooled to room temperature, and stored at −20°C until use. The total protein was separated by gel electrophoresis and transferred to a PVDF membrane. The primary antibody dilution ratios were as follows: NLRP3 (1:1000), ASC (1:1000), Cleaved caspase1 (1:1000), Cleaved Gasdermin D (1:1000), and β-Actin (1:10000). After incubation with HRP-labeled secondary antibodies diluted with TBST, immunoreactive bands were observed using a chemiluminescence method.

### 2.12 Immunohistochemical analysis of rat liver tissue

Rat liver tissue samples were sectioned, dewaxed, and subjected to antigen retrieval. The sections were treated with 3% H_2_O_2_ to inhibit endogenous peroxidase activity and then blocked with 5% normal goat serum at room temperature for 30 min to prevent non-specific binding signals. Diluted NLRP3 antibody (1:100) and GSDMD polyclonal antibody (1:100) were added as primary antibodies and incubated overnight at 4°C. After washing three times with PBS, HRP-labeled goat anti-rabbit/mouse secondary antibody was added and incubated at 37°C for 30 min. Freshly prepared DAB chromogenic solution and Mayer’s hematoxylin were then added for staining. The slides were washed with PBS, dehydrated, mounted, and observed and photographed under a biological microscope.

### 2.13 Immunofluorescence of HepG2 cells

HepG2 cells were washed with PBS and fixed with 4% paraformaldehyde. The cells were then permeabilized with 0.5% Triton X-100 (prepared in PBS) at room temperature and washed again with PBS. Goat serum was added to block antibodies at room temperature to reduce non-specific binding signals. Diluted NLRP3 antibody (1:100) and GSDMD polyclonal antibody (1:100) were added as primary antibodies and incubated overnight at 4°C. After washing three times with PBS, fluorescence (Cy3)-labeled goat anti-rabbit IgG (1:400) secondary antibody was added and incubated at 37°C for 1 h. Finally, DAPI was added for incubation for 5 min, and the cells were mounted and observed and photographed under a fluorescence microscope.

### 2.14 Statistical analysis

The data from these experiments are presented as means ± standard deviations (SDs). All experimental data were analyzed using Image-Pro Plus 6.0 and GraphPad Prism 8.0. For multiple comparisons, one-way analysis of variance (ANOVA) was used, followed by the Tukey test. A p-value <0.05 was considered statistically significant.

## 3 Results

### 3.1 Characterization of BSP

**FIGURE 4:**
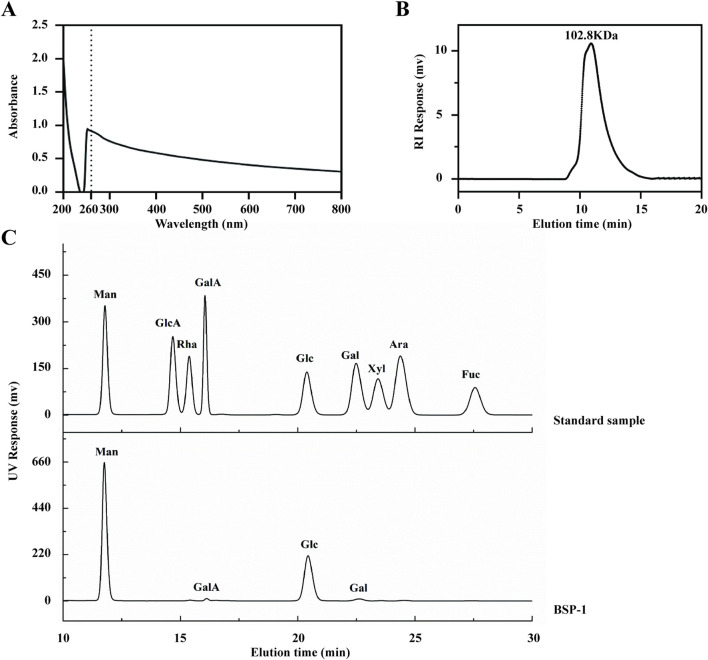
Characterization of BSP. **(A)** The UV Spectroscopy of BSP-1. **(B)** The average molecular weight of BSP-1. **(C)** The monosaccharide composition of BSP-1.

The purified BSP-1 was found to contain a polysaccharide content of 96.12% ± 0.08%, as determined by the phenol-sulfuric acid method. UV spectroscopy indicated that the composition was relatively homogeneous, with minimal other special impurities. The average molecular weight of BSP-1 was determined to be 102.8 kDa through high-performance gel permeation chromatography. HPLC analysis revealed that BSP is composed of mannose (Man), glucose (Glc), galactose (Gal), and galacturonic acid (GalA) in a molar ratio of 62.5:34.9:1.7:0.9 (Figures 4A–C).

### 3.2 Effect of BSP-1 on body weight and liver index in rats

To ascertain the impact of BSP-1 on MASLD *in vivo*, we observed that the body weights of rats in all groups gradually increased over time during the study period. Additionally, rats in the HFD group exhibited higher body weights and liver indices. However, after 4 weeks of BSP-1 intervention, the rate of weight gain was attenuated in all groups, with the most significant effect observed in the 400 mg/kg/d group. Anatomical observations showed that the livers of rats in the HFD group were more fragile and heterogeneous in texture, with a yellower color. Following BSP-1 intervention, the livers became smaller, redder, and showed a significant reduction in fatty degeneration (Figures 2B,C,E).

### 3.3 Effect of BSP-1 on liver tissue in rats and pathological tissue in HepG2 cells

Histopathological observations through H&E staining revealed severe hepatocyte degeneration and the formation of lipid vacuoles in MASLD rats from the HFD group. This phenomenon decreased with increasing concentrations of BSP-1 intervention (Figure 2E). Consequently, we used Oil Red staining to observe the lipid droplet content in the HFD-induced rat groups and FAA-induced HepG2 cell groups, further validating this observation (Figures 2E, 3I).

### 3.4 Effects of BSP-1 on biochemical indicators in rat serum and HepG2 cells

**FIGURE 5:**
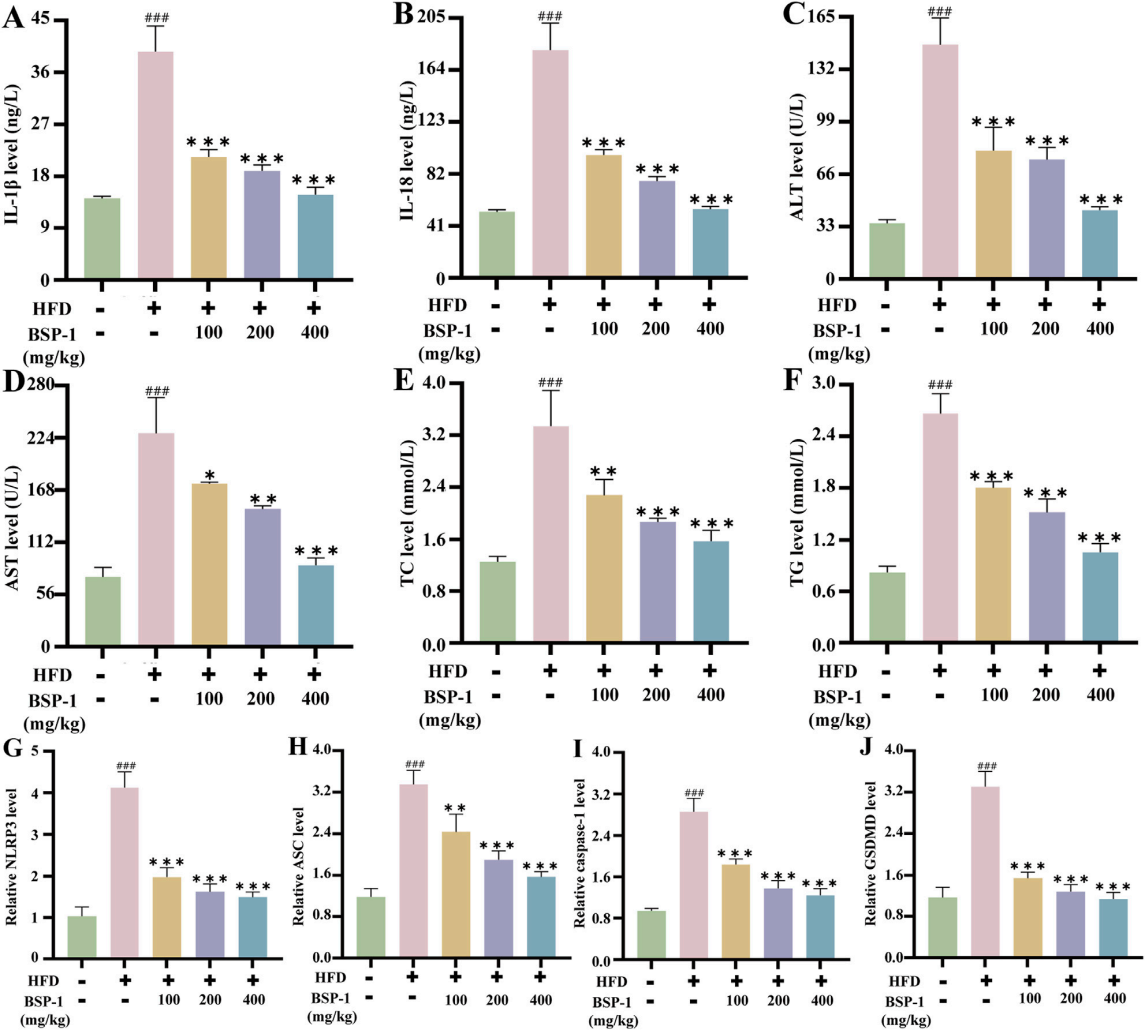
Measurement of IL-1β, IL-18, TC, TG, liver function in rat serum and qRT-PCR analysis in rat liver tissue. The measurement of **(A)** IL-1β **(B)** IL-18 **(C)** ALT **(D)** AST **(E)** TC **(F)** TG in rat serum. The mRNA levels of **(G)** the NLRP3 **(H)** the ASC **(I)** the caspase-1 **(J)** the GSDMD in rat liver tissue. The data are shown as the means ± SDs. ^#^ *p* < 0.05, ^##^ *p* < 0.01, ^###^ *p* < 0.001, vs. the control group; ^*^ *p* < 0.05, ^**^ *p* < 0.01, ^***^ *p* < 0.001, vs. the model group.

By measuring biochemical indicators in rat serum and HepG2 cells, the impact of BSP-1 on liver tissue damage and lipid deposition in MASLD was observed (Figures 3D–G, 5C–F). Compared with the control group, the levels of ALT, AST, TC, and TG in rat serum and HepG2 cells were significantly elevated in the model group. In contrast, the levels of these biochemical indicators were lower in the rat serum and HepG2 cells of the various BSP-1-treated groups compared to the model group. Notably, the reductions in ALT, AST, TC, and TG levels were most significant in the MASLD rat serum of the 400 mg/kg/d group and in the HepG2 cells of the FFA + BSP-1 (100 μg/mL) group, suggesting that BSP-1 is non-hepatotoxic and can improve HFD-induced liver tissue damage and lipid accumulation in rats, as well as FFA-induced liver damage and lipid accumulation in HepG2 cells.

### 3.5 Effects of BSP-1 on the expression of inflammatory cytokines in rat serum and HepG2 cells

**FIGURE 6:**
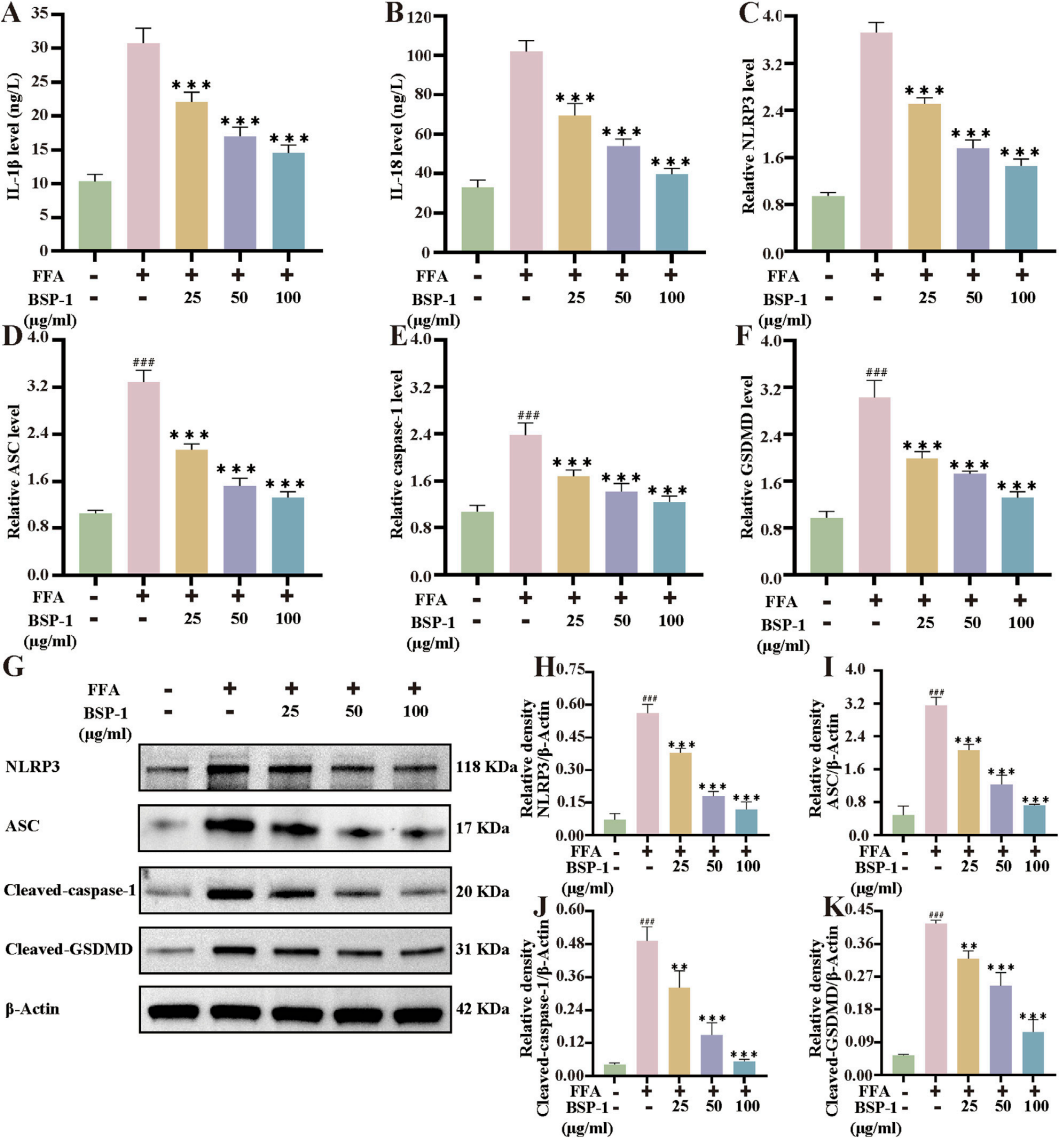
Measurement of IL-1β and IL-18, qRT-PCR and WB analysis in HepG2 cells. The measurement of **(A)** IL-1β and **(B)** IL-18 in HepG2 cells. The mRNA levels of **(C)** the NLRP3 **(D)** the ASC **(E)** the caspase-1 and **(F)** the GSDMD in HepG2 cells. **(G)** The WB analysis in HepG2 cells. **(H)** The NLRP3/β-Actin **(I)** the ASC/β-Actinof **(J)** the Cleaved-caspase-1/β-Actin and **(K)** the Cleaved-GSDMD/β-Actinof WB in HepG2 cells. The data are shown as the means ± SDs. ^#^ *p* < 0.05, ^##^ *p* < 0.01, ^###^ *p* < 0.001, vs. the control group; ^*^ *p* < 0.05, ^**^ *p* < 0.01, ^***^ *p* < 0.001, vs. the model group.

We then investigated the impact of BSP-1 on inflammatory cytokines in MASLD (Figures 5A,B, 6A,B). The levels of IL-1β and IL-18 in rat serum and HepG2 cells were significantly higher in the model group compared to the control group. However, after treatment with different doses of BSP-1, the levels of IL-1β and IL-18 in rat serum and HepG2 cells were significantly reduced compared to the model group, indicating that BSP-1 treatment can effectively decrease the production of inflammatory cytokines in HFD-induced rat serum and FFA-induced HepG2 cells.

### 3.6 Effects of BSP-1 on the pyroptosis-related NLRP3/caspase-1/GSDMD pathway in rat liver tissue and HepG2 cells

**FIGURE 7:**
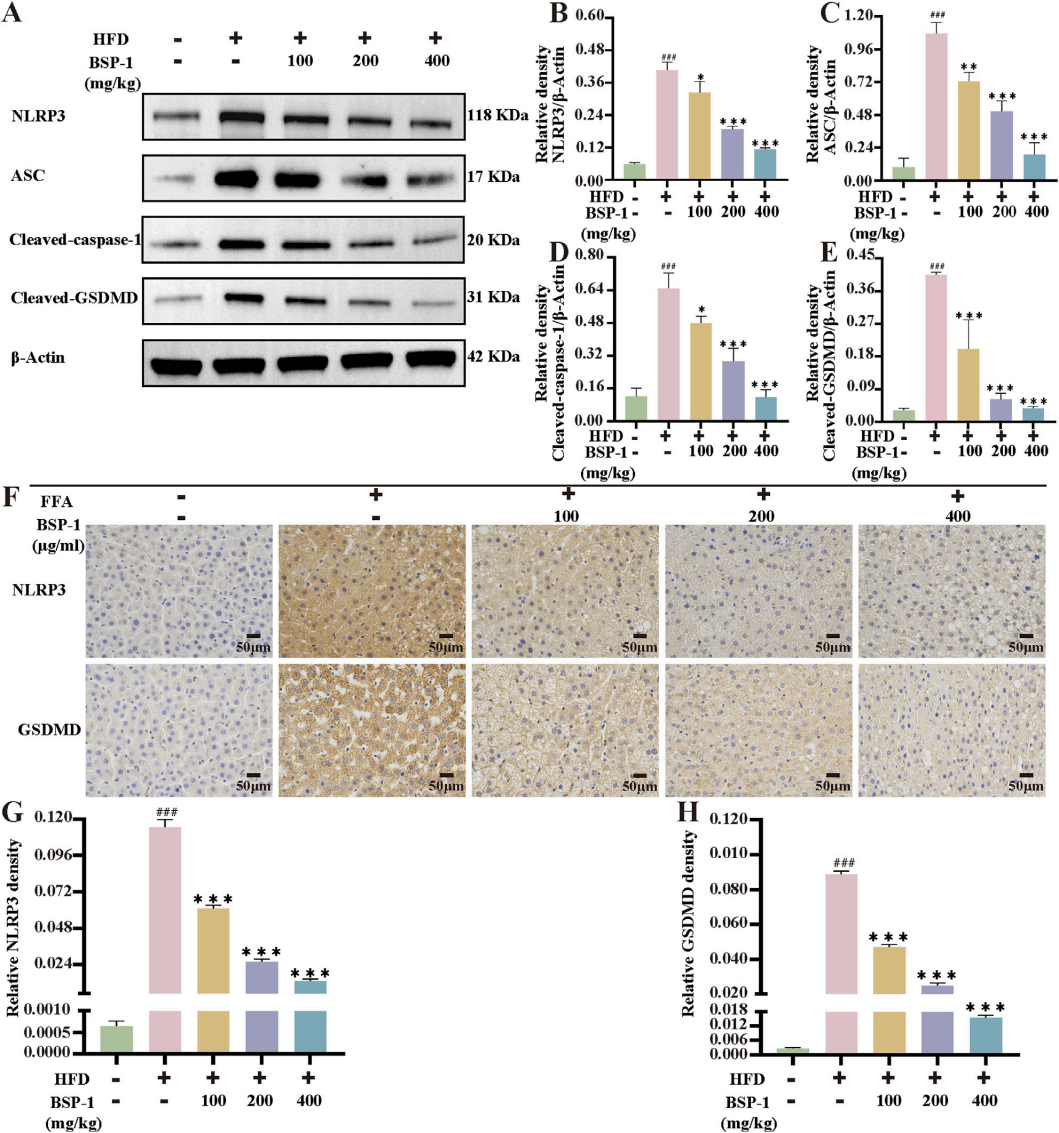
The WB analysis and immunohistochemical staining in rat liver tissue. **(A)** The WB analysis in rat liver tissue. **(B)** The NLRP3/β-Actin **(C)** the ASC/β-Actinof **(D)** the Cleaved-caspase-1/β-Actin and **(E)** the Cleaved-GSDMD/β-Actinof WB in rat liver tissue. **(F)** The evaluation of NLRP3 and GSDMD immunohistochemical staining (200 ×). The immunohistochemical density of **(G)** the NLRP3 and **(H)** the GSDMD in rat liver tissue. The data are shown as the means ± SDs. ^#^ *p* < 0.05, ^##^ *p* < 0.01, ^###^ *p* < 0.001, vs. the control group; ^*^ *p* < 0.05, ^**^ *p* < 0.01, ^***^ *p* < 0.001, vs. the model group.

**FIGURE 8:**
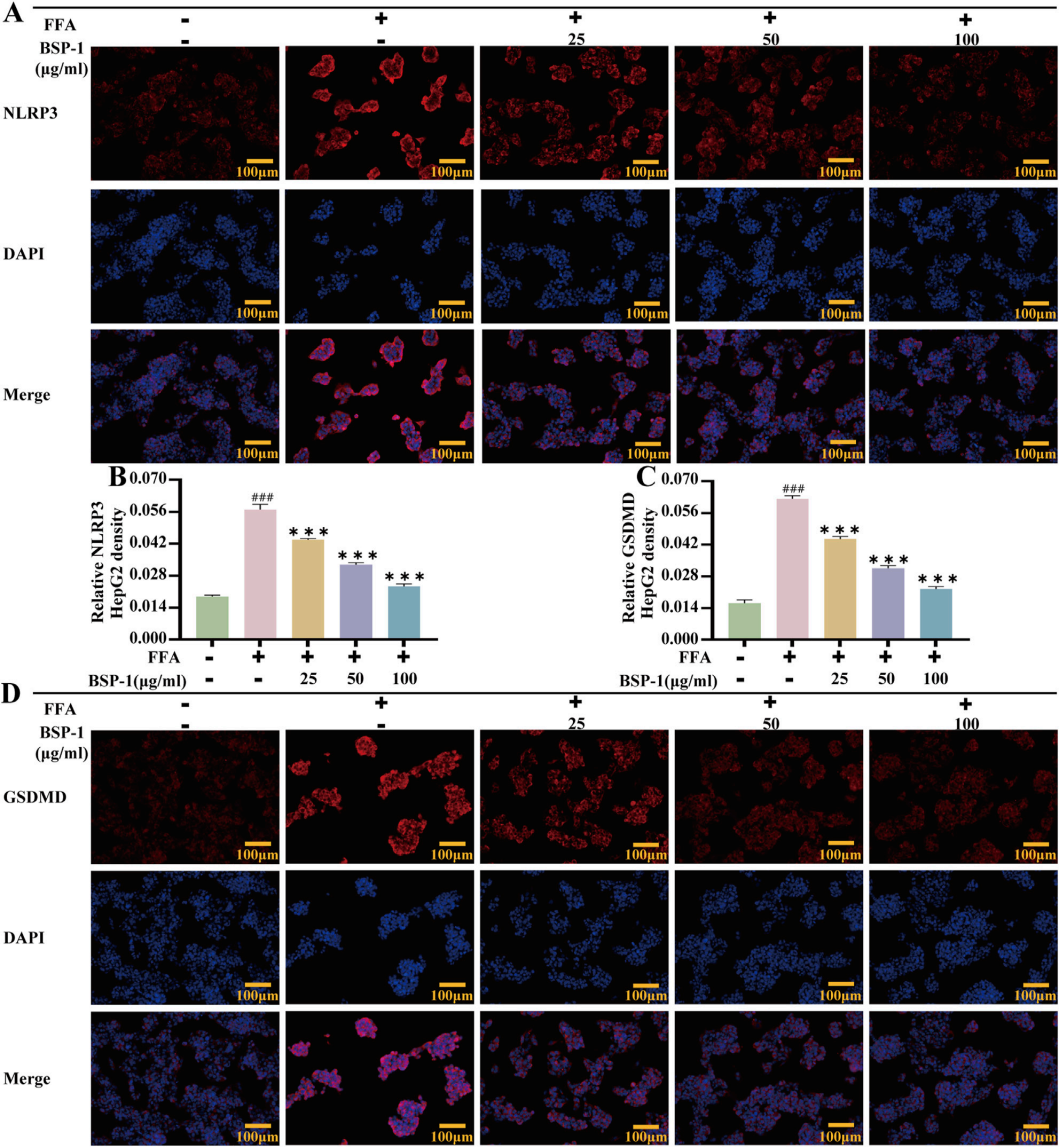
Immunofluorescence of HepG2 Cells. **(A)** The evaluation of NLRP3 immunofluorescence staining (100 ×). The immunohistochemical density of **(B)** the NLRP3 and **(C)** the GSDMD in HepG2 cells. **(D)** The evaluation of GSDMD immunofluorescence staining (100 ×). The data are shown as the means ± SDs. #*p* < 0.05, ##*p* < 0.01, ###*p* < 0.001, vs. the control group; *p < 0.05, **p < 0.01, ***p < 0.001, vs. the model group.

To further understand the effects of BSP-1 on MASLD through *in vivo* and *in vitro* experiments, we investigated the expression of proteins related to the pyroptosis-related NLRP3/caspase-1/GSDMD pathway. After WB detection and semi-quantitative analysis, the expression of NLRP3, ASC, Cleaved-caspase-1, and Cleaved-GSDMD was significantly increased in the model group compared to the normal group in both rat liver tissue and HepG2 cells. However, after intervention with the BSP-1 group, the expression of these related proteins was downregulated (Figures 6G–K, 7A–E). Additionally, immunohistochemical and immunofluorescence experiments, along with semi-quantitative analysis, were conducted to confirm this phenomenon (Figures 7F–H, 8A–D). To detect whether the relevant target genes were transcribed into mRNA, qRT-PCR analysis was performed, revealing a positive correlation between the expression trends of NLRP3, ASC, caspase-1, GSDMD-related mRNA and their protein levels in each group (Figures 5G–J, 6C–F).

## 4 Discussion

In recent years, polysaccharides have emerged as a well-known treasury of new pharmaceutical resources, possessing potential therapeutic effects on various diseases. BSP has good biocompatibility, degradability and low toxicity, and possesses excellent pharmacological activity and clinical application value (Zhu et al., 2023). Prior research has demonstrated that BSP ameliorates gut microbiota dysbiosis induced by a HFD in mice by reducing the Firmicutes/Bacteroidetes ratio, thereby mitigating abnormal weight gain (Zhang et al., 2024). Additionally, BSP exhibits the ability to alleviate hepatic fibrosis through the TLR2/TLR4-MyD88-NF-κB signaling pathway, ultimately achieving hepatic protection (Jiang et al., 2023). Although existing studies have shown that BSP has anti-inflammatory and liver-protecting effects, its specific therapeutic effect on MASLD has not been systematically studied. This study verified for the first time through *in vivo* and *in vitro* experiments the possibility of BSP-1 improving MASLD. To validate this hypothesis, we first extracted and purified the polysaccharide BSP-1 from the traditional Chinese medicine *Bletilla striata*. Subsequently, we induced MASLD in rats using a HFD and fatty degeneration in HepG2 cells with FFA, and administered BSP-1 to observe its regulatory effects on MASLD.

In the initial stages of our experiment, we extracted a novel neutral polysaccharide, BSP-1, which comprises Man, GalA, Glc, and Gal. Prior studies have demonstrated that polysaccharide-based therapeutics exhibit efficacy in improving MASLD. For instance, polysaccharides derived from Auricularia auricular-judae specifically target and activate the Toll-like receptor 4 (TLR4) on macrophages within the TLR4/NF-κB signaling pathway, which is situated upstream of the NLRP3 inflammasome. This activation enhances innate immune functions by promoting phagocytosis and cytokine production in macrophages (Tang et al., 2024). Similarly, the polysaccharide from *Gynostemma pentaphyllum*, upon degradation by intestinal microbiota, yields bioactive products that support the growth of beneficial gut flora such as *Akkermansia* and *Lactobacillus*. Concurrently, it downregulates the TLR2/NLRP3 pathway, thereby delaying the progression of MASLD in murine models (Yue et al., 2022). Furthermore, *Lycium barbarum* polysaccharide has been shown to mitigate liver injury in a methionine-choline deficient diet-induced steatohepatitis model by downregulating the NF-κB and NLRP3/6 pathways, thus exhibiting antioxidant and immunoregulatory properties (Xiao et al., 2018). Additionally, based on the monosaccharide composition of BSP, mannose intake has been reported to alleviate intrahepatic oxidative stress, inflammation, and fibrosis in thioacetamide-induced rats, thereby demonstrating hepatoprotective effects (Shaker et al., 2021). Besides monosaccharide composition, MW also significantly influences biological activity. The MW of BSP is 102.8 kDa. When compared to the MWs reported in previous studies, a lower MW is associated with reduced molecular volume, solubility, and viscosity, potentially indicating enhanced biological effects *in vivo* (Wang et al., 2019). Consequently, we further assessed the bioactivity of BSP concerning MASLD.

The disruption of the synthesis and excretion balance of free fatty acids and triglycerides in the liver leads to hepatic steatosis, which is crucial in the development of MASLD. The induction of HepG2 cells by FFA mixture with potential cytotoxicity and lipotoxicity (oleic acid to palmitic acid ratio of 2:1) represents an *in vitro* cell model that simulates benign chronic steatosis (Lee et al., 2019; Wang et al., 2022). SD rats fed a high-fat diet developed MASLD, accompanied by hepatic glycerol accumulation and hyperlipidemia. In our study, rats fed with HFD exhibited significant biochemical characteristics of MASLD, including hyperlipidemia and lipid accumulation in the liver. Significant lipid droplet aggregation was also observed in HepG2 cells induced by FFA. The non-specific clinical feature of MASLD is elevated liver transaminases (ALT and AST), which are positively correlated with most MASLD patients. Improving liver function damage and lipid metabolism disorders caused by MASLD has a positive effect on the treatment of MASLD. In this study, after treatment with BSP-1, elevated levels of TC, TG, and liver transaminases (ALT, AST) were significantly reduced, indicating that BSP-1 can alleviate liver cell damage caused by MASLD and improve lipid metabolism. Pathological section analysis also showed that BSP-1 treatment group showed a significant reduction in fat deposition and a alleviation of inflammatory infiltration. This further confirms the therapeutic effect of BSP-1 on MASLD and provides morphological evidence for subsequent molecular mechanism research.

**FIGURE 9:**
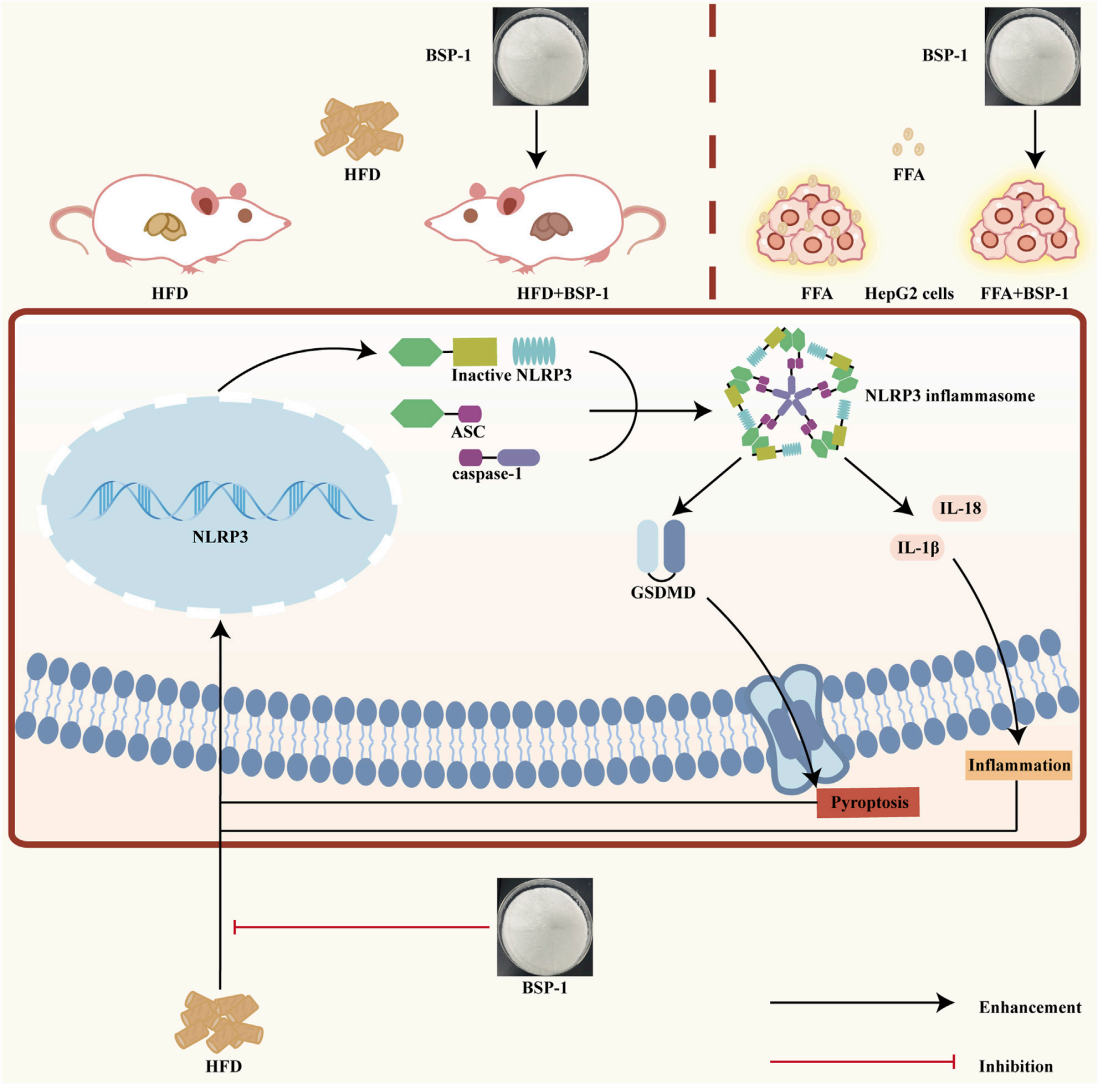
The impact of BSP-1 on MASLD by evaluating its ability to reduce the expression of pyroptosis-related NLRP3/caspase-1/GSDMD pathway in both HFD-induced rat models *in vivo* and FFA-induced HepG2 cells *in vitro*.

MASLD is closely related to chronic inflammation of the liver. The NLRP3 inflammasome is essential for the progression of chronic inflammation and has recently become a potential new therapeutic target for MASLD (Chan et al., 2023; Hutchison et al., 2023). It is ubiquitously present in hepatic immune and parenchymal cells and effectively recognizes disturbances caused by PAMPs and DAMPs in the hepatic cellular milieu, thereby exerting innate immune functions (Sharma and Kanneganti, 2021). In general, the levels of the sensor NLRP3, the adapter ASC, and the effector caspase-1 within the multi-molecular protein complex NLRP3 inflammasome remain relatively stable and low. Although immune cells, upon sensing PAMPs and DAMPs, activate the NF-κB signaling pathway to increase the expression of NLRP3 genes, thereby elevating their numbers, post-translational modifications such as ubiquitination, phosphorylation, and sumoylation, along with interactions between NLRP3 and organelles, further regulate immune cells to prevent easy activation of the NLRP3 inflammasome in response to stimulation (Swanson et al., 2019). Once activated in macrophage- and monocyte-dominant liver sites, the NLRP3 inflammasome triggers the production of pro-inflammatory cytokines, lysosomal damage, and increased reactive oxygen species, further inducing the transcription of NLRP3 genes. Additionally, the prion-like polymerization of fibrils formed during the docking of the PYD domains of NLRP3 and ASC facilitates the recruitment of caspase-1 by the caspase recruitment domain of ASC. The activated NLRP3 inflammasome mainly induces pyroptosis by cleving GSDMD, allowing its N-terminal domain to bind to the cell membrane and form pores. Meanwhile, it promotes the secretion of extracellular active pro-inflammatory cytokines IL-1β and IL-18 (Hooftman et al., 2020), which intensifies the liver inflammatory response and accelerates the development of MASLD. In our study, we found that BSP-1 could effectively weaken the protein and mRNA expressions of NLRP3, ASC, caspase-1 and GSDMD, indicating that the NLRP3 inflammasome-related pathway was inhibited. In the rat model induced by HFD, immunohistochemical analysis also showed that BSP-1 could improve the expression of NLRP3 and GSDMD proteins in liver tissues. The same conclusion was reached by immunofluorescence detection of the expression of NLRP3 and GSDMD proteins in the HepG2 cells model induced by FFA. Furthermore, BSP-1 also inhibits the production of pro-inflammatory cytokines IL-1β and IL-18 in the HFD-induced rat and FFA-induced HepG2 cells, which further indicates its inhibitory effect on liver inflammation. Therefore, BSP-1 plays a role in alleviating MASLD by preventing steatosis, inflammation, and pyroptosis, which may be relate to the inhibition of NLRP3/caspase-1/GSDMD signaling pathway (Figure 9).

## 5 Conclusion

In conclusion, our research results show that BSP-1 can improve liver injury and steatosis caused by MASLD in both *in vitro* and *in vivo* experiments, this effect may be attributed to the regulation of the pyrother-related NLRP3/caspase-1/GSDMD pathway. This result provides evidence that BSP-1 has the potential to treat MASLD. However, the existing studies are still unclear whether BSP-1 exerts its effect through direct action on NLRP3 or other indirect mechanisms. In the future, the interaction mechanism between it and the NLRP3/caspase-1/GSDMD pathway should be further clarified. Meanwhile, further clarifying the molecular structure of BSP-1 is of great significance for studying its pharmacological activity and molecular mechanism. Clinical trials based on human MASLD patients will be carried out in the future to verify the therapeutic potential of BSP-1. Therefore, as an emerging polysaccharide drug, BSP-1 still requires more in-depth and comprehensive research in the process of MASLD research.

## Data Availability

The original contributions presented in the study are included in the article/Supplementary Material, further inquiries can be directed to the corresponding authors.
