# Supplementary material for: Bletilla striata polysaccharides ameliorate metabolic-associated fatty liver disease by decreasing the NLRP3 inflammasome and pyroptosis
Source: Front Pharmacol. 2025 Jun 20;16:1563275. doi: 10.3389/fphar.2025.1563275 (PMC12231352; doi:10.3389/fphar.2025.1563275)
Supplement: Supplementary file 1 [file DataSheet1.PDF]

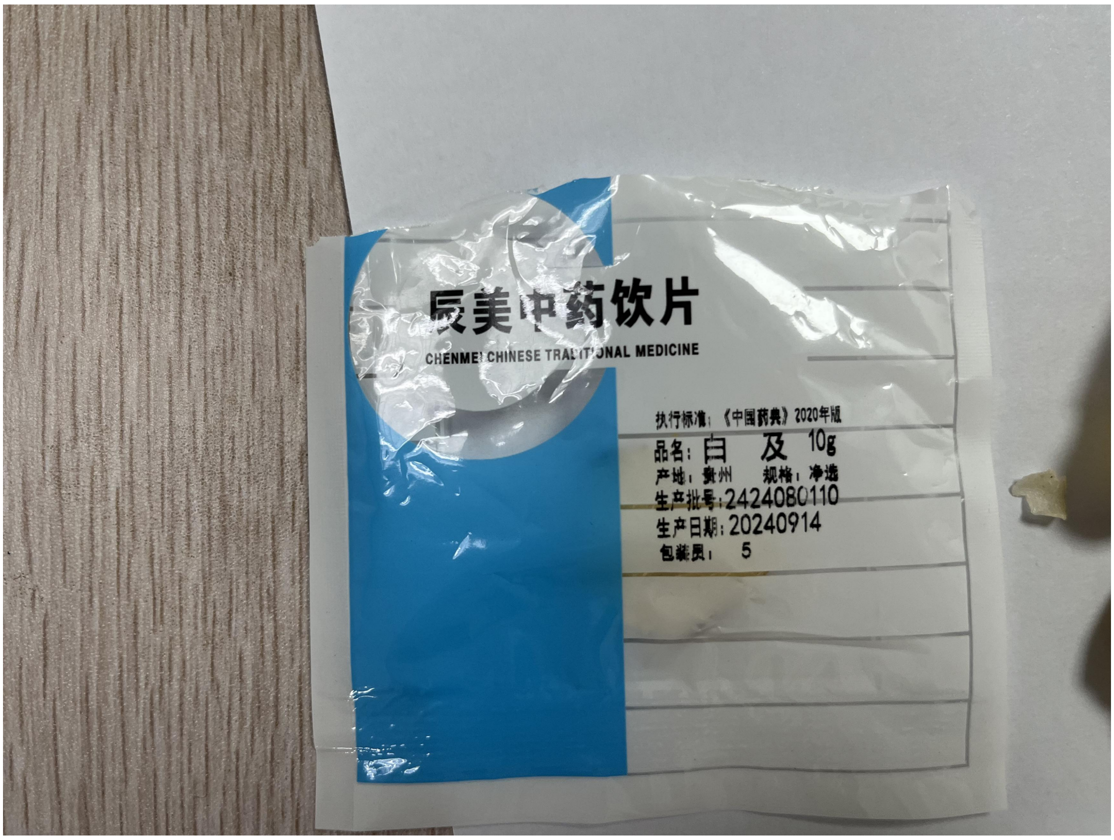

Bletilla striata (Thunb.) Rchb.f. tubers from Hubei University of Traditional Chinese Medicine

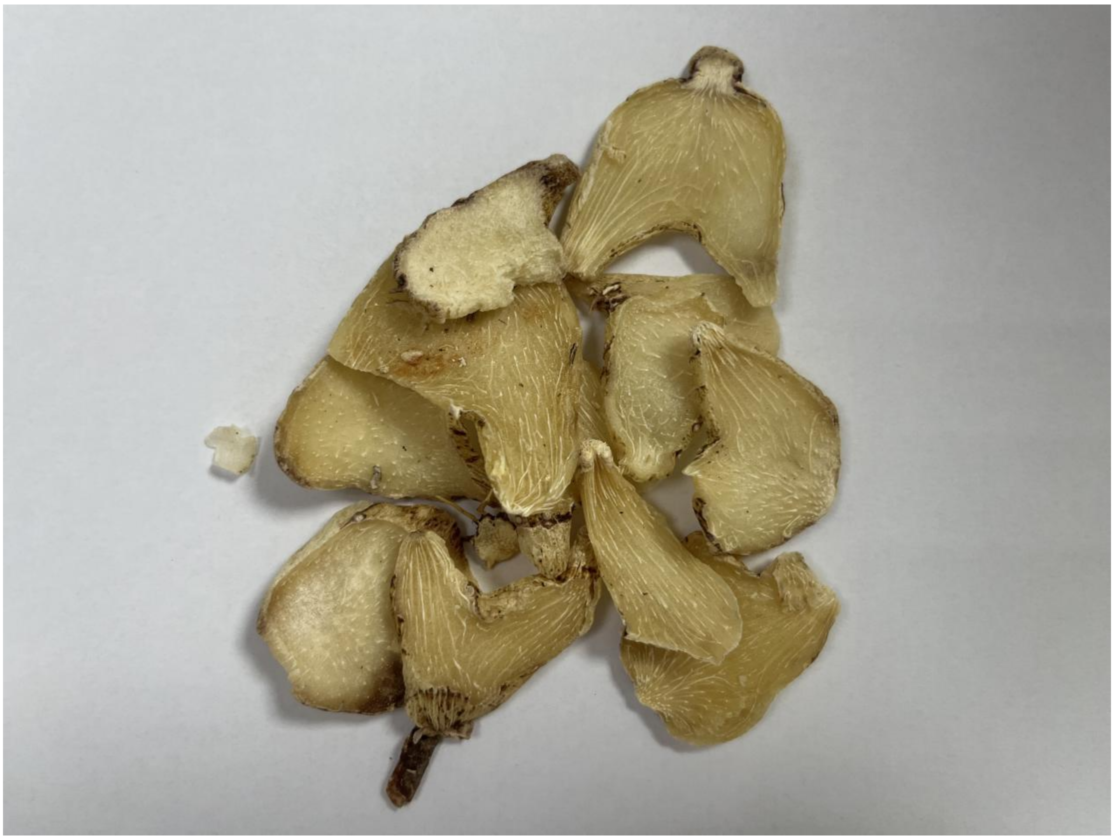

Bletilla striata (Thunb.) Rchb.f. tubers

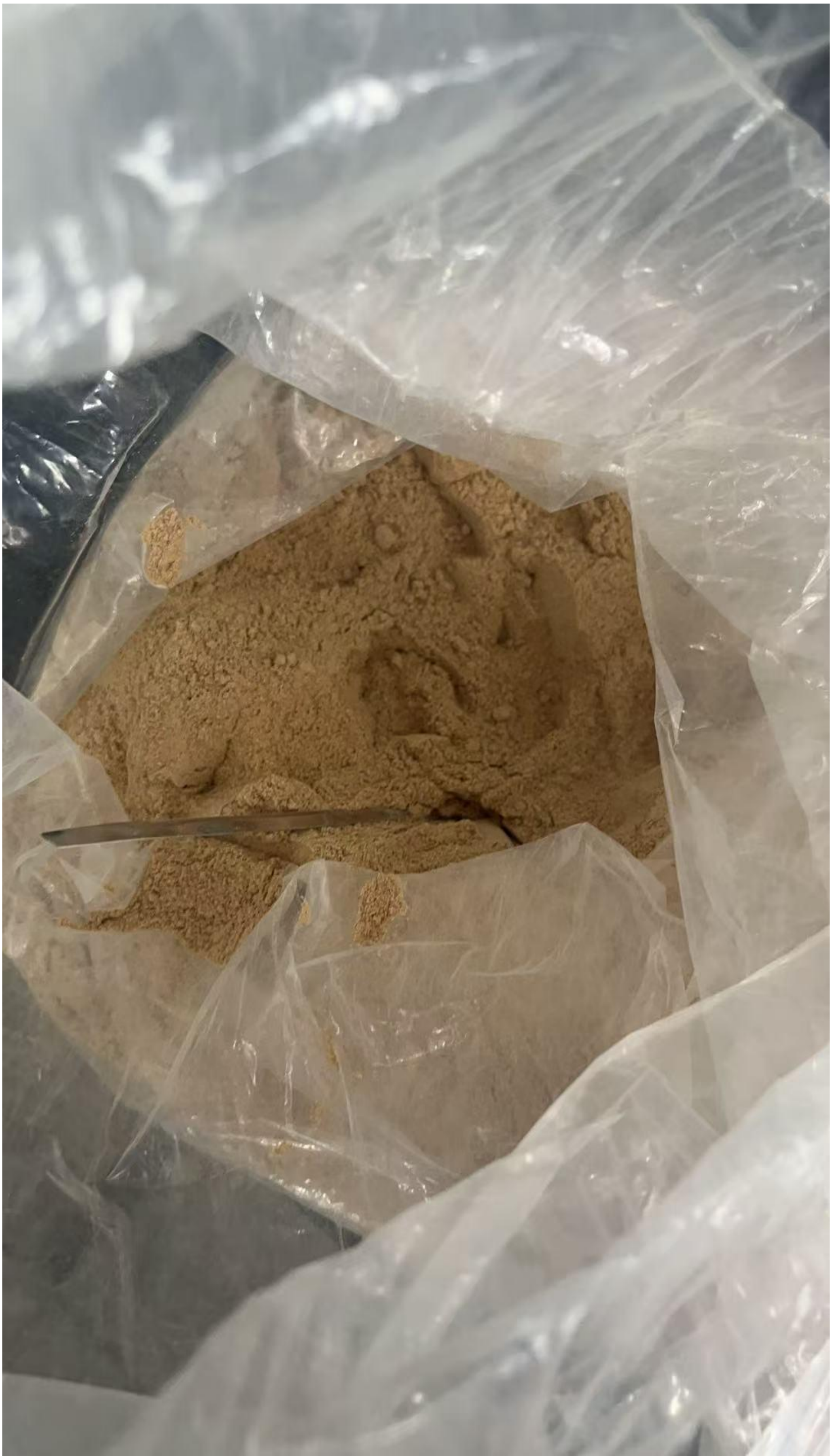

Bletilla striata (Thunb.) Rchb.f. tubers powder
